# Supplementary material for: Gender discrimination in hiring: An experimental reexamination of the Swedish case
Source: PLoS One. 2021 Jan 29;16(1):e0245513. doi: 10.1371/journal.pone.0245513 (PMC7845993; doi:10.1371/journal.pone.0245513)
Supplement: S1 File — (PDF) [file pone.0245513.s001.pdf]

Gender Discrimination in Hiring: An Experimental

Reexamination of the Swedish Case

Table A1 presents the results of regressing each covariate included in the complete model specification used in the main paper (column 6 of Table 2). The lack of statistical significance (beyond the chance of spurious results) across the estimates indicates that our treatment variable was independent of observed covariates. Of course, there is always an untestable possibility that there are unobservables which are not independent. But given the randomization procedure used for all three data collections we consider this less likely.

Table A1: Ordinary Least Squares Estimates of Balance of Covariates

Covariate category	(1)	(2)	(3)	(4)	(5)
Skills	Experience	Experience^2	Computer	Language	Active
	-0.001	-0.000	-0.005	0.008	-0.015
	(0.004)	(0.000)	(0.023)	(0.024)	(0.024)
Occupations	Store clerk	Vehicle mechanic	Cleaner	Enrolled nurse	Customer service
	-0.003	0.028	-0.034	-0.017	-0.036
	(0.045)	(0.035)	(0.026)	(0.036)	(0.070)
	Waitstaff	Telemarketing	Preschool teacher	Chef (REF)	Childcare
	-0.006	0.076	-0.009	0.039	-0.014
	(0.024)	(0.076)	(0.031)	(0.027)	(0.060)
	Truck/Delivery driver	Warehouse worker	IT developer	B2B sales	Accounting clerk
	-0.012	0.049	-0.024	0.076*	-0.058
	(0.029)	(0.043)	(0.041)	(0.041)	(0.040)
Occupation level	Gender ratio	Male median wage	Female median wage	Median wage difference	
	-0.050	0.058	0.042	-0.430*	
	(0.032)	(0.060)	(0.066)	(0.255)	
Vacancy level	Full time	Contract length	Urban		
	0.036*	0.023	0.018		
	(0.020)	(0.019)	(0.018)		

Note: Covariates regressed on treatment variable (male dummy). Robust standard errors in parentheses. *, **, ***, indicate rejection of the null hypothesis at the 10, 5, and 1 percent significance levels, respectively. “REF” is short for reference and indicates that Chef jobs were the reference category when using fixed effects.

Table A2 shows that the main discrimination estimates from the paper are robust to the Heckman-Siegelman critique. As “Male-level (Variance)” was not significant in any sub-sample, we conclude that differences in the variance of unobservables between male and female applicants did not affect results.

Table A2: Neumark's method of testing Heckman-Siegelman critique

	(1)	(2)	(3)	(4)
Panel A: All three studies	Full sample	Male dominated occupations	Mixed occupations	Female dominated occupations
Probit				
Male	-0.053***	0.045	-0.021	-0.154***
	(0.016)	(0.033)	(0.027)	(0.027)
Heteroskedastic probit				
Male	-0.056***	0.048	-0.026	-0.156***
	(0.017)	(0.034)	(0.028)	(0.028)
Male-level (marginal)	-0.035	0.029	0.017	-0.137***
	(0.028)	(0.048)	(0.045)	(0.046)
Male-level (variance)	-0.020	0.019	-0.043	-0.019
	(0.026)	(0.041)	(0.041)	(0.041)
Tests				
S.D ratio of unobservables (Male/Female)	0.895	1.111	0.802	0.899
Test S.D. ratio = 1	0.402	0.661	0.236	0.622
Overidentification test	0.986	0.957	0.871	0.983
LR test: Probit vs. Heteroskedastic Probit	0.427	0.645	0.283	0.641
Observations	3254	845	1211	1198
	(5)	(6)	(7)	(8)
Panel B: Study 3 only	Full sample	Male dominated occupations	Mixed occupations	Female dominated occupations
Probit				
Male	-0.036	0.069	0.012	-0.214***
	(0.031)	(0.060)	(0.048)	(0.058)
Heteroskedastic probit	.			
Male	-0.030	0.086	0.008	-0.214***
	(0.031)	(0.054)	(0.048)	(0.058)
Male-level (marginal)	-0.068*	0.014	0.036	-0.217***
	(0.041)	(0.073)	(0.059)	(0.084)
Male-level (variance)	0.039	0.072	-0.028	0.004
	(0.034)	(0.045)	(0.039)	(0.065)
Tests				
S.D ratio of unobservables (Male/Female)	1.445	2.214	0.768	1.031
Test S.D. ratio = 1	0.313	0.185	0.389	0.956
Overidentification test	0.832	0.890	0.969	0.998
LR test: Probit vs. Heteroskedastic Probit	0.219	0.031	0.445	0.956
Observations	1071	303	456	312

Note: This table reports the results of Neumark's method for addressing the Heckman-Siegelman critique of correspondence studies. All models include skill controls, vacancy controls, study controls, and occupation fixed effects. Panel A includes all three studies, while panel B only includes Study 3 where skill controls were independently randomized.

Table A3 shows the interactions relevant to taste-based discrimination discussed briefly in the main paper.

Table A3: Linear probability models with interactions

	(1)	(2)	(3)	(4)	(5)
All occupations					
Treatment					
Male	-0.051***	0.012	-0.056**	-0.025	-0.053
	(0.016)	(0.027)	(0.022)	(0.030)	(0.040)
Interactions					
High CI		0.123***			0.102***
		(0.025)			(0.034)
Male × High CI		-0.092***			0.046
		(0.033)			(0.045)
Female contact				0.081**	0.083**
				(0.033)	(0.033)
Male × Female contact				-0.063	-0.069
				(0.045)	(0.045)
Observations	3,252	3,252	1,619	1,619	1,619
Occupation FE	No	No	No	No	No
Vacancy controls	Yes	Yes	Yes	Yes	Yes
Skill controls	Yes	Yes	No	No	No
Gender ratio control	Yes	Yes	Yes	Yes	Yes
Sample	All studies	All studies	Study 1 & 2	Study 1 & 2	Study 1 & 2

Note: This table reports the interaction effects between being a male applicant and customer interaction and having the application evaluated by a female. Linear probability models were used. Robust standard errors in parentheses. *, **, ***, indicate rejection of the null hypothesis at the 10, 5, and 1 percent significance levels, respectively. “FE” is short for fixed effects

During data collection, any time a response was received which was difficult to classify as either positive or negative we coded it according to our best judgement but also indicated this ambiguity in a dummy variable which we called a “maybe” response. All other analysis here and in the main paper uses these best judgements for the outcome variable, where out of 157 maybe responses 106 were considered positive and 51 as negative. Tables A4 and A5 shows how recoding all edge case responses as either all positive or all negative affects the results reported in Table 3 in the main paper. The former constitutes a more lenient definition of what should be considered a positive callback, while the latter constitutes a less lenient

definition. As we can see, compared to results in Table 3 point estimates naturally change, but none of our conclusions change under either paradigm. Another, perhaps more straightforward way to show that classification of responses was not dependent on gender is to run the regression with maybe response as the outcome variable and treatment as the dependent variable, doing so shows no significant connection (LPM: $\beta = .008, p = .287$).

Table A4: Edge cases coded as positive

		(1)	(2)	(3)	(4)
		All occupations	Male dominated occupations	Mixed occupations	Female dominated occupations
All studies					
	Male	-0.052***	0.042	-0.025	-0.146***
		(0.016)	(0.032)	(0.026)	(0.026)
	Constant	0.348***	0.564***	0.370***	0.382***
		(0.068)	(0.185)	(0.101)	(0.134)
	Observations	3,252	844	1,210	1,198
Study 1					
	Male	-0.069***	0.006	-0.071	-0.110**
		(0.027)	(0.055)	(0.045)	(0.043)
	Constant	0.520***	0.475***	0.508***	0.161
		(0.089)	(0.180)	(0.143)	(0.111)
	Observations	1,049	255	381	413
Study 2					
	Male	-0.053**	0.053	-0.017	-0.142***
		(0.026)	(0.056)	(0.043)	(0.040)
	Constant	0.504***	0.295**	0.741***	0.069
		(0.082)	(0.144)	(0.129)	(0.100)
	Observations	1,132	286	373	473
Study 3					
	Male	-0.036	0.060	0.009	-0.205***
		(0.029)	(0.054)	(0.045)	(0.055)
	Constant	0.461***	0.254	0.447***	0.493***
		(0.081)	(0.157)	(0.114)	(0.159)
	Observations	1,071	303	456	312
Occupation FE		Yes	Yes	Yes	Yes
Job controls		Yes	Yes	Yes	Yes
Skill controls		Yes	Yes	Yes	Yes

Note: This table reports the marginal effect of being a male applicant in occupations with different gender ratios using linear probability models when recoding all 157 edge case responses as positive. Robust standard errors in parentheses. *, **, ***, indicate rejection of the null hypothesis at the 10, 5, and 1 percent significance levels, respectively. Occupation fixed effects, skill controls, and job controls were included in each model, i.e. specifications in line with column 6 of Table 2.

Table A5: Edge cases coded as negative

	(1)	(2)	(3)	(4)
	All occupations	Male dominated occupations	Mixed occupations	Female dominated occupations
All studies				
Male	-0.045***	0.018	-0.008	-0.124***
	(0.015)	(0.030)	(0.025)	(0.025)
Constant	0.337***	0.595***	0.349***	0.295**
	(0.067)	(0.182)	(0.101)	(0.130)
Observations	3,252	844	1,210	1,198
Study 1				
Male	-0.056**	-0.017	-0.051	-0.084**
	(0.025)	(0.047)	(0.042)	(0.041)
Constant	0.377***	0.336**	0.358***	0.139
	(0.085)	(0.132)	(0.132)	(0.107)
Observations	1,049	255	381	413
Study 2				
Male	-0.060**	0.012	-0.007	-0.140***
	(0.024)	(0.051)	(0.040)	(0.037)
Constant	0.336***	0.215	0.653***	-0.040
	(0.076)	(0.140)	(0.123)	(0.089)
Observations	1,132	286	373	473
Study 3				
Male	-0.016	0.059	0.031	-0.162***
	(0.029)	(0.053)	(0.045)	(0.054)
Constant	0.456***	0.322**	0.431***	0.429***
	(0.080)	(0.158)	(0.114)	(0.154)
Observations	1,071	303	456	312
Occupation FE	Yes	Yes	Yes	Yes
Job controls	Yes	Yes	Yes	Yes
Skill controls	Yes	Yes	Yes	Yes

Note: This table reports the marginal effect of being a male applicant in occupations with different gender ratios using linear probability models when recoding all 157 edge case responses as negative. Robust standard errors in parentheses. *, **, ***, indicate rejection of the null hypothesis at the 10, 5, and 1 percent significance levels, respectively. Occupation fixed effects, skill controls, and job controls were included in each model, i.e. specifications in line with column 6 of Table 2.

As skills were only varied for Study 3, we test for statistical discrimination only with those data. Figure A6 shows the interaction with work experience and our treatment variable graphically. There is tentative but insignificant evidence of a decline in discrimination as work experience increases. The weakness of this result could be due to our skill variables, in this case work experience, having a weak

effect on positive response rates overall. Or it could be that work experience is not a factor upon which employers statistically discriminate. Another way to look for statistical discrimination is using a linear probability model (LPM) and an F-test of joint significance with the interactions of all skill variables used in Study 3. Again, we found no significant evidence of statistical discrimination, $F(5,1056) = .83$, $p = .526$.

Figure A6: Interaction between work experience and male treatment in Study 3

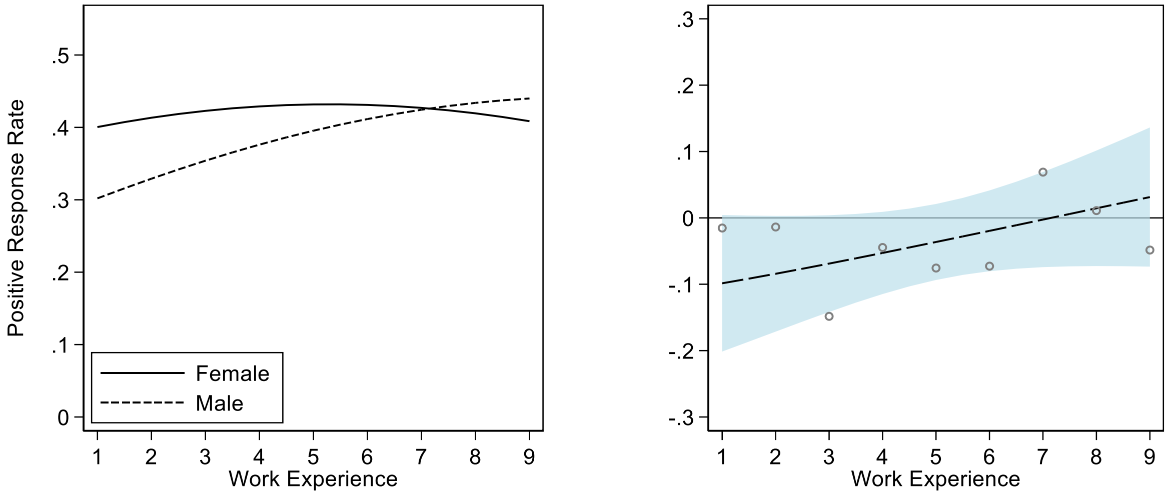

Note: These graphs are based on probit estimates of the interaction between male applicant and work experience. The left-hand graphs show the predicted probability of a positive response for applicants given their work experience. The right-hand graph plots the differences in predicted probability of a positive response, and the circles indicate the raw mean differences in positive responses by work experience. The estimates behind these graphs only use only data from Study 3 as work experience was not independently varied in Studies 1 & 2.

Table A7 shows the probit estimates behind the graphs in Fig 1 in the main paper. Column 1 of Table A7 is used for the top two graphs of Fig 1 and column 2 of Table A7 is used for the bottom two graphs. LPM estimates are shown in the bottom panel of Table A7 to show that they are similar.

Table A7: Models with interactions for Fig 1

	(1)	(2)	(3)
All occupations - Probit			
Treatment			
Male	0.214**	-0.275***	0.096
	(0.103)	(0.068)	(0.121)
Interactions			
Female workforce	-1.082**	0.009	-1.101**
	(0.479)	(0.094)	(0.482)
Male × Female workforce	-0.683***		-0.631***
	(0.170)		(0.172)
Wage difference	-0.032	0.209**	0.050
	(0.060)	(0.089)	(0.099)
Male × Wage difference		-0.214**	-0.159*
		(0.084)	(0.085)
Constant	-0.648***	-0.574***	-0.595***
	(0.186)	(0.175)	(0.190)
Observations	3,101	3,101	3,101
All occupations - LPM			
Treatment			
Male	0.076**	-0.094***	0.034
	(0.035)	(0.023)	(0.039)
Interactions			
Female workforce	-0.337**	0.002	-0.345**
	(0.159)	(0.033)	(0.161)
Male × Female workforce	-0.236***		-0.221***
	(0.059)		(0.058)
Wage difference	-0.007	0.072**	0.020
	(0.020)	(0.032)	(0.036)
Male × Wage difference		-0.075**	-0.060**
		(0.030)	(0.030)
Constant	0.259***	0.288***	0.279***
	(0.067)	(0.064)	(0.068)
Observations	3,101	3,101	3,101
Occupation FE	No	No	No
Vacancy controls	Yes	Yes	Yes
Skill controls	Yes	Yes	Yes
Sample	All studies	All studies	All studies

Note: This table reports the interaction effects between being a male applicant and applying to a job in a female dominated occupation and the interaction with the median occupational wage difference. Probit models were used in the first panel and are the ones underlying Fig 1, in the second panel linear probability model (LPM) estimates are reported. Standard errors in parentheses (robust standard errors were used for LPM estimates). *, **, ***, indicate rejection of the null hypothesis at the 10, 5, and 1 percent significance levels, respectively. B2B sales was excluded as an outlier to aid readability of graphical representation in Fig 1. To aid interpretation of these estimates, the wage difference variable was divided by 1000. "FE" is short for fixed effects.
