# Supplementary material for: Gender discrimination in hiring: An experimental reexamination of the Swedish case
Source: PLoS One. 2021 Jan 29;16(1):e0245513. doi: 10.1371/journal.pone.0245513 (PMC7845993; doi:10.1371/journal.pone.0245513)
Supplement: Attachment — Submitted filename: PONE_D_20_3157_reviewer_report.pdf [file pone.0245513.s002.pdf]

Reviewer's Report on PLOS ONE manuscript no. PONE-D-20-31057

Gender discrimination in hiring: An experimental reexamination of the Swedish case.

By Ali Ahmed, Mark Granberg, and Shantanu Khanna

Summary.

The authors recycle three earlier studies on discrimination by correspondence tests in order to compile a new dataset that enables to investigate the extent of gender discrimination in first replies to written job applications.

More specifically, they use the 'non–treated' cases of their earlier studies on hiring discrimination against ex–offenders (Ahmad and Långe, 2017), crime victims (Ahmad and Långe, 2019), and transgenders (Granberg, Andersson, and Ahmed 2020). Among these 'non–treated' (those not belonging to the group of ex–offenders, crime victims, respectively transgenders) there are males and females.

Using a linear probability model, the authors find a significant overall negative effect of being a male on the probability of a positive response of 5 percentage points (ppt). This results from the combination of a significant negative effect of 14 ppt in female dominated occupations (that is, occupations with more than two thirds female employees), an insignificant negative effect of 1.7 ppt in mixed occupations (between one third and two thirds females), and an insignificant positive effect of 4 ppt in male dominated occupations. The results are robust after control for skill variation, job type (full time, length of contract, urban), occupation, and the study from which the observation was drawn.

Preceding to the analysis, the authors investigate whether the resumes of different gender are randomly distributed over the other characteristics, a necessary requirement for their design (see further). Furthermore, spurious effects due to different standard deviations of the unobservables (see Heckman and Siegelman, 1993) are shown to be precluded.

An attempt is made to make the distinction between statistical discrimination and taste discrimination. Finally, a discussion about the evolution of gender inequality in occupations in connection with the obtained results follows.

Discussion.

– One of the most salient properties of this study is that it does not follow the pairwise matching approach, but resumes of treated (men) and non–treated (women) are

randomly sent out. In this way, the authors meet a recent critique on the pairwise matching approach (Phillips, 2019). The main point of Phillips (2019) is that the probability of receiving a positive answer does not only depends on the applicant's own qualities, but also on those of the other candidates. By pairwise matching, the experimental design affects the latter, what is not the case if only one application is sent out per vacancy. I am not particularly convinced that the random assignment of one application per vacancy overcomes the difficulty. Indeed, the pool of other candidates is then not affected, but their qualities may differ across vacancies. This is only one of many examples of possible vacancy specific effects which might bias the estimates of the coefficient of interest. Not only for this reason, but also conceptually, I still much appreciate the pairwise matching approach. Indeed, in my view, discrimination cannot appropriately be measured by differences in positive responses. Discrimination is an unequal treatment of people differing only in one particular trait. There is an indication of possible discrimination of gender *e.g.* if two equivalent candidates that do differ only in gender, are treated unequally (*e.g.* one is invited for an interview, while the other not). Both being invited or both being not invited, are signals of equal treatment. I don't know whether such an alternative analysis of pairwise matched data would be subject to the same criticism as the one Phillips (2019) raised against the analysis of differences in the the probability of a positive response. I presume it does, but still then there is a trade–off between the potential vacancy specific effect bias, and the pool of other applicants' bias. I do not expect the authors to follow my point of view and to change their analysis, but some deeper motivation of their particular stance is expected for a scientific article.

– The authors provide a discussion of the meaning of their findings for the gender imbalance in certain professions. On the one hand, p.14 discusses the results of a probit regression of the response on the percentage of females in the occupation. On the other hand, the Discussion section is devoted to the possible consequences of gender discrimination in (the first step of) hiring for the persistence of gender imbalance in certain professions. For the first part, reporting the regression results and exact functional forms (for example in the Supplementary material) would be welcomed. For example, it is not clearly specified which 'interactions' are taken up (I guess with the male dummy).

  The combination of both considerations might raise the question whether female

domination in certain occupations is due to discrimination, or whether female domination in certain professions leads to discriminating hiring behaviour. I find the Discussion section and the empirical material in that section too premature for a scientific article. I would therefore advocate to drop that section, and discuss more in depth the regression results reported on p.14 by means of the two graphs in the top of Fig. 1, and only briefly mention there the problem of potential reverse causality.

Minor points.

- The discussion on the gender wage gap in the introduction seems to me not relevant for the subject of this paper.
- Apart from the distribution of the applications across the occupations, and the gender distribution of the responses per occupation, few information about the sample is given. The gender distribution across occupations of the resumes sent out, should be added to Table 1 or in the Supplementary material. Importantly, information about the age mentioned in the resumes is indispensable, as it may be an important confounding factor (Lahey, 2008).
- The Heckman–Siegelman critique is mentioned on p.14, and test results are reported in Table A2 of the Supplementary material, but nowhere it is appropriately explained what was done (*e.g.* What do the reported coefficients for 'male (marginal)'and 'male (variance)', the latter being negative, mean?) and why the results allow to reject the possibility of spurious correlation.
- The choice between a linear probability model for the main analysis and probits for some of the additional analyses, is poorly motivated.
- In the Supplementary material on p.6, the authors mention that skills were only varied for the third study from which they drew their data. Still, they report in Table 2 coefficients for the skill variables for the whole sample. Does it imply that these variables are constant for a large part of the data? Can it explain limited significance? Again, this point shows that there is only limited information on the raw data in the paper.

References.

Ahmed A.M. & Lång E. (2017), "The employability of ex–offenders: a field experiment in the Swedish labor market," *IZA Journal of Labor Policy* **6**, 6(2017), doi:10.1186/s40173-017-0084-2.

Ahmed A.M. & Lång E. (2019), "Victimized Twice: A Field Experiment on the Employability of Victims," *Victims & Offenders.* Vol.14(7), 859–874.

Granberg M., Andersson P.A., & Ahmed A. (2020), "Hiring Discrimination Against Transgender People: Evidence from a Field Experiment,"*Labour Economics*, Vol. 65, doi:10.1016/j.labeco.2020.10186.

Heckman J.J. & Siegelman P. (1993), " The Urban Institute audit studies: their methods and findings," in: M. Fix & R.J. Struyk (Eds.), *Clear and Convincing Evidence. Measurement of Discrimination in America.* Washington D.C.: The Urban Institute Press, 187–275.

Lahey J.N. (2008), "Age, Women, and Hiring: An Experimental Study," *Journal of Human Resources*, Vol.43(1), 30–56.

Phillips D.C. (2019), "Do Comparisons of Fictional Applicants Measure Discrimination When Search Externalities are Present? Evidence from Existing Experiments, *The Economic Journal*, Vol.129(Issue 621), 2240–2264.
