# Supplementary material for: Gender discrimination in hiring: An experimental reexamination of the Swedish case
Source: PLoS One. 2021 Jan 29;16(1):e0245513. doi: 10.1371/journal.pone.0245513 (PMC7845993; doi:10.1371/journal.pone.0245513)
Supplement: Attachment — Submitted filename: PONE-D_31057 review.pdf [file pone.0245513.s003.pdf]

The paper estimates gender discrimination in hiring combining data from three large-scale field experiments in Sweden. They find that men have lower hiring probabilities overall, and especially when applying for a female-dominated occupation.

While some evidence on gender based hiring discrimination exists even for Sweden, it is true that this evidence has not been totally conclusive. The correspondence experiments used in the paper are appropriate to measure this discrimination and seem well-designed and executed. Moreover, the paper is well-written. I nevertheless have some comments which I believe the authors should address.

1. The authors go very quickly over a number of key concepts, making the paper difficult to read for anyone not familiar with the literature or methodology used.
    a. Apart from one sentence (p. 2 line 54), the authors do not explain what a correspondence experiment is, why it is an appropriate method to measure hiring discrimination, or what it’s main advantages and disadvantages are as opposed to other methods.
    b. On p. 15, line 311, the authors talk about statistical discrimination without defining this theory. The same is true or taste-based discrimination further in the document.
2. Further on page 15, the authors state “In our case, skills were only weakly associated with positive employer rates in general.”. It is unclear from which analysis the authors draw this conclusion. This entire paragraph is confusion and the reader lacks information in order to understand the conclusions drawn here.
    a. Moreover, as statistical discrimination occurs when employers make assumptions about a candidate’s skills following imperfect information, it is more about the quantity of the skill-related signals than the quality. This is something the authors cannot test for, but should be acknowledged.
3. The paper misses a “methodology” section. Parts of the method are explained in the “Data” section and others in the “Results” part, making the structure of the paper difficult to follow
