# Supplementary material for: Gender discrimination in hiring: An experimental reexamination of the Swedish case
Source: PLoS One. 2021 Jan 29;16(1):e0245513. doi: 10.1371/journal.pone.0245513 (PMC7845993; doi:10.1371/journal.pone.0245513)
Supplement: Attachment — Submitted filename: Response_to_Reviewers.docx [file pone.0245513.s004.docx]

**Reviewer comments: Gender discrimination in hiring: An experimental reexamination of the Swedish case**

**Editor: Stijn Baert**

Dear Dr. Granberg,

Thank you for submitting your manuscript to PLOS ONE. After careful consideration, we feel that it has merit but does not fully meet PLOS ONE’s publication criteria as it currently stands. Therefore, we invite you to submit a revised version of the manuscript that addresses the points raised during the review process.

I agree with the reviewers that this article is a sound contribution and that after revision work, in line with the reviewers' comments, there is a high probability that it will be suitable for publication in PLOS ONE.

From my side, I would ask you to acknowledge a bit more recent field experimental research (published the last five years) on hiring discrimination against females. My register can guide you in this respect: <https://users.ugent.be/~sbaert/research_register.htm>.

In addition, it would be great if you could propose a communication strategy to maximise the article's scientific ànd societal impact upon publication.

Please submit your revised manuscript by Dec 17 2020 11:59PM. If you will need more time than this to complete your revisions, please reply to this message or contact the journal office at plosone@plos.org. When you're ready to submit your revision, log on to https://www.editorialmanager.com/pone/ and select the 'Submissions Needing Revision' folder to locate your manuscript file.

Please include the following items when submitting your revised manuscript:

•	A rebuttal letter that responds to each point raised by the academic editor and reviewer(s). You should upload this letter as a separate file labeled 'Response to Reviewers'.

•	A marked-up copy of your manuscript that highlights changes made to the original version. You should upload this as a separate file labeled 'Revised Manuscript with Track Changes'.

•	An unmarked version of your revised paper without tracked changes. You should upload this as a separate file labeled 'Manuscript'.

**Response to Editor**

We thank the editor for his fair and reasoned evaluation of our manuscript. In response to the first comment about references we have amended the section about previous studies to include both, as the editor suggested, more recent work but also added references to previous work which was previously lumped into “eight published correspondence studies on gender discrimination in hiring”. We have also included the suggested link to the correspondence test repository.

As for the communication strategy, here follows a number of steps we will take, should the paper be accepted:

1. We will work with our competent communications department here at Linköping University and inform them about our publication. They in turn will communicate our results through our home page, Twitter, Instagram, and Facebook. They typically also do a press release in both Swedish and English meaning that our results will be communicated to both national and international press outlets.
2. We will work to disseminate these results in our own research network, specifically within a national Swedish collaboration on research in labor market discrimination supported by the Swedish Research Council.
3. Furthermore, we will share our work at seminars both at Linköping University and University of California, Irvine.

**Reviewer 1: Bart Capéau**

**From email:**

I find the analysis in this paper neatly done. I have two problems though. First, in its present state, the choices made by the authors are poorly motivated. Second, too few information on the raw sample is given, and this is in my view indispensable for this type of research.

In my reviewer report in attachment, I give more details on these points.

**From file:**

The authors recycle three earlier studies on discrimination by correspondence tests in order to compile a new dataset that enables to investigate the extent of gender discrimination in ﬁrst replies to written job applications.

More speciﬁcally, they use the ‘non–treated’ cases of their earlier studies on hiring discrimination against ex–oﬀenders (Ahmad and Långe, 2017), crime victims (Ahmad and Långe, 2019), and transgenders (Granberg, Andersson, and Ahmed 2020). Among these ‘non–treated’ (those not belonging to the group of ex–oﬀenders, crime victims, respectively transgenders) there are males and females.

Using a linear probability model, the authors ﬁnd a signiﬁcant overall negative eﬀect of being a male on the probability of a positive response of 5 percentage points (ppt). This results from the combination of a signiﬁcant negative eﬀect of 14 ppt in female dominated occupations (that is, occupations with more than two thirds female employees), an insigniﬁcant negative eﬀect of 1.7 ppt in mixed occupations (between one third and two thirds females), and an insigniﬁcant positive eﬀect of 4 ppt in male dominated occupations. The results are robust after control for skill variation, job type (full time, length of contract, urban), occupation, and the study from which the observation was drawn.

Preceding to the analysis, the authors investigate whether the resumes of diﬀerent gender are randomly distributed over the other characteristics, a necessary requirement for their design (see further). Furthermore, spurious eﬀects due to diﬀerent standard deviations of the unobservables (see Heckman and Siegelman, 1993) are shown to be precluded.

An attempt is made to make the distinction between statistical discrimination and taste discrimination. Finally, a discussion about the evolution of gender inequality in occupations in connection with the obtained results follows.

**Major points**

1. One of the most salient properties of this study is that it does not follow the pairwise matching approach, but resumes of treated (men) and non–treated (women) are randomly sent out. In this way, the authors meet a recent critique on the pairwise matching approach (Phillips, 2019). The main point of Phillips (2019) is that the probability of receiving a positive answer does not only depends on the applicant’s own qualities, but also on those of the other candidates. By pairwise matching, the experimental design aﬀects the latter, what is not the case if only one application is sent out per vacancy. I am not particularly convinced that the random assignment of one application per vacancy overcomes the diﬃculty. Indeed, the pool of other candidates is then not aﬀected, but their qualities may diﬀer across vacancies. This is only one of many examples of possible vacancy speciﬁc eﬀects which might bias the estimates of the coeﬃcient of interest. Not only for this reason, but also conceptually, I still much appreciate the pairwise matching approach. Indeed, in my view, discrimination cannot appropriately be measured by diﬀerences in positive responses. Discrimination is an unequal treatment of people diﬀering only in one particular trait. There is an indication of possible discrimination of gender e.g. if two equivalent candidates that do diﬀer only in gender, are treated unequally (e.g. one is invited for an interview, while the other not). Both being invited or both being not invited, are signals of equal treatment. I don’t know whether such an alternative analysis of pairwise matched data would be subject to the same criticism as the one Phillips (2019) raised against the analysis of diﬀerences in the the probability of a positive response. I presume it does, but still then there is a trade–oﬀ between the potential vacancy speciﬁc eﬀect bias, and the pool of other applicants’ bias. I do not expect the authors to follow my point of view and to change their analysis, but some deeper motivation of their particular stance is expected for a scientiﬁc article.
2. The authors provide a discussion of the meaning of their ﬁndings for the gender imbalance in certain professions. On the one hand, p.14 discusses the results of a probit regression of the response on the percentage of females in the occupation. On the other hand, the Discussion section is devoted to the possible consequences of gender discrimination in (the ﬁrst step of) hiring for the persistence of gender imbalance in certain professions. For the ﬁrst part, reporting the regression results and exact functional forms (for example in the Supplementary material) would be welcomed. For example, it is not clearly speciﬁed which ‘interactions’ are taken up (I guess with the male dummy).

**Minor points**

1. The discussion on the gender wage gap in the introduction seems to me not relevant for the subject of this paper.
2. Apart from the distribution of the applications across the occupations, and the gender distribution of the responses per occupation, few information about the sample is given. The gender distribution across occupations of the resumes sent out, should be added to Table 1 or in the Supplementary material. Importantly, information about the age mentioned in the resumes is indispensable, as it may be an important confounding factor (Lahey, 2008).
3. The Heckman–Siegelman critique is mentioned on p.14, and test results are reported in Table A2 of the Supplementary material, but nowhere it is appropriately explained what was done (e.g. What do the reported coeﬃcients for ‘male (marginal)’and ‘male (variance)’, the latter being negative, mean?) and why the results allow to reject the possibility of spurious correlation.
4. The choice between a linear probability model for the main analysis and probits for some of the additional analyses, is poorly motivated.
5. In the Supplementary material on p.6, the authors mention that skills were only varied for the third study from which they drew their data. Still, they report in Table 2 coeﬃcients for the skill variables for the whole sample. Does it imply that these variables are constant for a large part of the data? Can it explain limited signiﬁcance? Again, this point shows that there is only limited information on the raw data in the paper.

**Response to Reviewer 1**

**Major points**

1. We acknowledge that views differ on this point, and we appreciate the opportunity to expand on our reasoning around matched pair testing in correspondence tests. We have done so in the extended data section which is now titled “Experimental Method and Data” (see pages 6-8). We think our position on the matter is made clearer by the additions, and thank reviewer 1 for his input which, we think, greatly improved our argumentation on the issue in this paper.
2. We agree that this was not presented properly in the previous version and have clarified exactly which interaction is being referred to (see page 16). We now inform the reader that probit estimates are available in table A7 of the supplementary material (which we added). We have also added LPM-estimates to table A7 to show that they are similar to probit estimates. In going over this analysis we found a small error in our code, which marginally changed the look of our graphed interaction effects in this particular instance, though nothing of substance changed we thank reviewer 1 for prompting us to go over this analysis again to catch the minor error.

**Minor points**

1. Our intention in the introduction was to only briefly mention gender wage gaps in order to motivate the importance of occupational segregation, and the contribution of the latter to the former. We find it to be an important part of motivating why we should care about gender discrimination in the labor market, and to provide important contextual information. We have looked over our writing on this in the hopes of clarifying this view.
2. We have added a note in the method section on the age of applicants being 28 in all three studies. As for the other information requested, it is available in Table 1. The first panel titled gender ratio provides the share of female workers in each of the occupations for 2016 and 2019. As regards the balance of male and female CVs sent to each occupation, this share is calculable from the information in the table and is shown to not be an issue by the balance of covariates tests in Table A1 in the supplementary material. We are quite limited in what additional information would fit in Table 1, and therefore hope that this explanation will be sufficient.
3. We have expanded on this, hopefully it is a lot clearer now (Page 15-16).
4. Yes, hopefully this is clearer now as we have added some discussion about this in the remade method and data section.
5. Yes, this is very possible, and we now discuss this in the paper. We also further emphasize now that skills were only varied in study 3 in the additions on page 17.

**Reviewer 2: Eva Van Belle**

The paper estimates gender discrimination in hiring combining data from three large-scale field experiments in Sweden. They find that men have lower hiring probabilities overall, and especially when applying for a female-dominated occupation.
While some evidence on gender based hiring discrimination exists even for Sweden, it is true that this evidence has not been totally conclusive. The correspondence experiments used in the paper are appropriate to measure this discrimination and seem well-designed and executed. Moreover, the paper is well-written. I nevertheless have some comments which I believe the authors should address.

1. The authors go very quickly over a number of key concepts, making the paper difficult to read for anyone not familiar with the literature or methodology used.
   a. Apart from one sentence (p. 2 line 54), the authors do not explain what a correspondence experiment is, why it is an appropriate method to measure hiring discrimination, or what it’s main advantages and disadvantages are as opposed to other methods.
   b. On p. 15, line 311, the authors talk about statistical discrimination without defining this theory. The same is true or taste-based discrimination further in the document.
2. Further on page 15, the authors state “In our case, skills were only weakly associated with positive employer rates in general.”. It is unclear from which analysis the authors draw this conclusion. This entire paragraph is confusion and the reader lacks information in order to understand the conclusions drawn here.
   a. Moreover, as statistical discrimination occurs when employers make assumptions about a candidate’s skills following imperfect information, it is more about the quantity of the skill-related signals than the quality. This is something the authors cannot test for, but should be acknowledged.
3. The paper misses a “methodology” section. Parts of the method are explained in the “Data” section and others in the “Results” part, making the structure of the paper difficult to follow

**Response to Reviewer 2**

1. We have tried to expand on these concepts
   a. We agree this was too sparsely explained in the submitted version and have expanded the description, especially in the extended data section which is now titled “Experimental Method and Data”. Specifically, the second paragraph in this section explains in greater detail what correspondence studies are, and how they are superior to other regression-based approaches to measuring discrimination (which are not causal).
   b. We agree statistical discrimination came up rather suddenly as presented in the submitted version. We therefore now briefly introduce the concept in the introduction; but as this analysis is not a focus of the paper, we feel that we do not want to take up too much space in the introduction by discussing well-established concepts in the literature on discrimination.
2. We have tried to clarify this section and made explicit reference to the specific table and column in which the weakness of our skill variables is shown.
   a. We understand that it is not possible to satisfactorily determine whether statistical discrimination is more about the quantity of the skill-related signals as opposed to quality. The theory, as proposed, does not make this distinction, and we remain agnostic about that in the paper as well..
3. Yes, we agree this was not optimally executed. Our challenge was that really the “method” for this study is in the combining the different data sets and thus we focused on their similarities and differences. We have expanded our description of the experimental approach, and moved some of the method-parts away from the results section We feel that combining the experimental method and data sections is a reasonable compromise as describing the data is so tightly interconnected with the method. We hope that the revised section is clearer.

**General Response**

We thank the editor, Stijn Baert, and the two referees, Bart Capéau and Eva Van Belle, for their invaluable input. In our view, their comments and perspectives definitely helped us produce a significantly improved article.
